# Supplementary material for: MetaRibo-Seq measures translation in microbiomes
Source: Nat Commun. 2020 Jun 29;11:3268. doi: 10.1038/s41467-020-17081-z (PMC7324362; doi:10.1038/s41467-020-17081-z)
Supplement: Supplementary file 10 — Supplementary Data 7 [file 41467_2020_17081_MOESM10_ESM.zip › File2/Confidence_High_Taxonomy/187083_out.krona.html]

Javascript must be enabled to view this page.

members
magnitude
magnitudeUnassigned
count
unassigned
taxon
rank

187083\_out

84

superkingdom
84
2

phylum
84
201174

class
84
84998

83
order
84999

family
83
84107

83
5
102106

SRS015431\_contig\_number\_contig-100\_5706.441817SRS020233\_contig\_number\_58284SRS049773\_contig\_number\_27704SRS143070\_contig\_number\_contig-100\_12241.87013SRS144714\_contig\_number\_contig-100\_14932.96591
genus

74426

SRS011084\_contig\_number\_7203SRS011084\_contig\_number\_contig-100\_2910.194178SRS012849\_contig\_number\_18908SRS013098\_contig\_number\_34624SRS014683\_contig\_number\_15839SRS014923\_contig\_number\_contig-100\_14843.103617SRS015217\_contig\_number\_14272SRS015663\_contig\_number\_contig-100\_3904.169295SRS018313\_contig\_number\_contig-100\_1751.34912SRS018351\_contig\_number\_contig-100\_241.63523SRS020328\_contig\_number\_contig-100\_1227.177265SRS023715\_contig\_number\_24263SRS042966\_contig\_number\_14490SRS047014\_contig\_number\_contig-100\_4257.130768SRS047433\_contig\_number\_contig-100\_16508.16509SRS049896\_contig\_number\_14068SRS049896\_contig\_number\_contig-100\_193.204223SRS050752\_contig\_number\_contig-100\_4852.4852SRS050998\_contig\_number\_11271SRS053356\_contig\_number\_contig-100\_4592.261879SRS053398\_contig\_number\_contig-100\_7826.46701SRS065504\_contig\_number\_26314SRS075878\_contig\_number\_20308SRS077194\_contig\_number\_10197SRS077335\_contig\_number\_9536SRS077392\_contig\_number\_25203SRS077392\_contig\_number\_40702SRS077392\_contig\_number\_contig-100\_9689.220259SRS097920\_contig\_number\_3020SRS104400\_contig\_number\_42899SRS143876\_contig\_number\_contig-100\_247.208863SRS144362\_contig\_number\_3939SRS147557\_contig\_number\_26623SRS148511\_contig\_number\_11937SRS148874\_contig\_number\_13327SRS893259\_contig\_number\_contig-100\_2327.37489
36
species

2292334

SRS012849\_contig\_number\_contig-100\_0.183088
1
species

2
species
2292324

SRS023583\_contig\_number\_contig-100\_31654.31654SRS065176\_contig\_number\_contig-100\_33357.33358

species
15

SRS013687\_contig\_number\_34581SRS014235\_contig\_number\_46220SRS017103\_contig\_number\_32665SRS019068\_contig\_number\_119352SRS045645\_contig\_number\_contig-100\_29643.29643SRS076929\_contig\_number\_contig-100\_35156.81152SRS098655\_contig\_number\_contig-100\_19001.19002SRS098881\_contig\_number\_contig-100\_11590.61121SRS1054691\_contig\_number\_contig-100\_4858.4859SRS1055056\_contig\_number\_contig-100\_7322.46295SRS143342\_contig\_number\_contig-100\_13923.91124SRS143466\_contig\_number\_29759SRS148424\_contig\_number\_contig-100\_6037.148496SRS892882\_contig\_number\_contig-100\_8070.50308SRS893259\_contig\_number\_contig-100\_1613.1614
742722

species
1

SRS018351\_contig\_number\_contig-100\_562.113316
2292014

species
4

SRS013687\_contig\_number\_contig-100\_16507.137220SRS017433\_contig\_number\_contig-100\_7766.46160SRS050026\_contig\_number\_contig-100\_9867.46206SRS078665\_contig\_number\_contig-100\_17841.53786
2292028

2292023

SRS023914\_contig\_number\_22926
1
species

2292024

SRS020233\_contig\_number\_2326SRS049773\_contig\_number\_15737
2
species


SRS021484\_contig\_number\_596
2292316
species
1

2292330

SRS015431\_contig\_number\_86729SRS017521\_contig\_number\_contig-100\_1376.264291SRS020869\_contig\_number\_19373SRS065504\_contig\_number\_contig-100\_1838.195124SRS144135\_contig\_number\_8882
5
species

1
species
1896993

SRS1041091\_contig\_number\_12357

species
1

SRS144362\_contig\_number\_19835
2292013

species
1

SRS020233\_contig\_number\_47872
2292208


SRS147557\_contig\_number\_34027SRS893378\_contig\_number\_contig-100\_23593.23594
2292011
species
2

2292026

SRS063518\_contig\_number\_contig-100\_5484.199051
1
species

1262850

SRS019030\_contig\_number\_contig-100\_14392.14392SRS050422\_contig\_number\_contig-100\_5179.163452SRS077194\_contig\_number\_contig-100\_14065.52871SRS893373\_contig\_number\_contig-100\_15835.15836
4
species

1643822
order
1

1
family
1643826

84108
1
genus

572010

SRS148159\_contig\_number\_54433
1
species
